# Correlations between core muscle strength endurance and upper-extremity performance in adolescent male sub-elite handball players

**DOI:** 10.3389/fspor.2022.1050279

**Published:** 2022-11-10

**Authors:** Julian Bauer, Markus Gruber, Thomas Muehlbauer

**Affiliations:** ^1^Division of Movement and Training Sciences/Biomechanics of Sport, University of Duisburg-Essen, Essen, Germany; ^2^Department of Sport Science, Human Performance Research Centre, University of Konstanz, Konstanz, Germany

**Keywords:** trunk, shoulder mobility/stability, throwing velocity, young athletes, performance

## Abstract

Handball is an Olympic contact sport with high physical, tactical, and technical demands by the players. Out of the different techniques, throwing is the most important one to be able to score. The objective of the study was to investigate the relationships between core muscle strength endurance (Bourban test: ventral, dorsal and lateral chain) and shoulder mobility/stability (Upper Quarter Y Balance test [YBT-UQ]) as well as throwing velocity in adolescent male sub-elite handball players (*N* = 32, age: 17.1 ± 0.7 years, height: 181.8 ± 6.3 cm, BMI: 24.6 ± 4.9 kg/m^2^). All participants were free of injuries at least two weeks prior to the study, experienced (training experience: 8.5 ± 3.3 years) handball players who were tested mid-season in the evening of one of their training sessions. Pearson correlations were calculated for core muscle strength endurance with (a) shoulder mobility/stability and (b) throwing velocity. The throwing arm reach displayed significant correlations (both *p* < 0.05) between the Bourban test (ventral chain) and the inferolateral reach direction (IL) of the YBT-UQ (*r* = 0.41) as well as the composite score (CS) (*r* = 0.34). For the dorsal chain, significant correlations (all *p* < 0.01) were found for the medial (MD) (*r* = 0.42) and IL (*r* = 0.61) reach direction as well as the CS (*r* = 0.51). For the right but not the left side of the lateral chain, significant correlations (both *p* < 0.05) were detected for the IL reach direction (*r* = 0.40) and the CS (*r* = 0.35). For the non-throwing arm reach, significant correlations were found between the ventral chain and the MD reach direction (*r* = 0.53, *p* < 0.01) as well as the CS (*r* = 0.31, *p* < 0.05). For the dorsal chain, significant correlations (all *p* < 0.01) were found for the MD (*r* = 0.47) and IL (*r* = 0.44) reach direction as well as the CS (*r* = 0.41). For the lateral chain, significant correlations were detected for the MD (left and right side: *r* = 0.49, *p* < 0.01) and IL (left and right side: *r* = 0.35, *p* < 0.05) reach direction as well as the CS (left and right side: *r* = 0.37, *p* < 0.05). The dorsal chain but not the ventral and lateral chain of the Bourban test showed a significant correlation with throwing velocity (*r* = 0.33, *p* < 0.05). Our results indicate that better core muscle strength endurance is associated with better shoulder mobility/stability as well as partially higher throwing velocity of adolescent male sub-elite handball players. Therefore, practitioners should integrate core muscle strength endurance exercises into the training routines to improve upper-extremity performance of this target group.

## Introduction

Handball is a popular sport worldwide which places complex demands on the players in terms of technical, tactical, and physical characteristics (1). Substantial differences in terms of anthropometric features as well athletic performance parameters such as throwing and sprinting are reported between playing positions and competitive levels of handball players (2–4). One example of the complex interplay of these different factors is throwing which is the most important technique in handball as a higher throwing velocity increases the likelihood to score (5, 6). Due to the proximal-to-distal sequence of developing force while throwing (7, 8) a well-developed core strength is necessary. In this context, the core is often referred to as the kinetic link (9) which enables dynamic activities of the extremities by providing proximal stability for distal mobility (10). Additionally, good shoulder mobility and stability is needed (11) as the acceleration of the ball is also influenced by a wide elbow extension and shoulder internal rotation with the necessity of a maximal external rotation (12). An effective kinetic chain during throwing is therefore characterized by strength, flexibility, power generation, a well-developed efficient task specific muscle activation as well as a sequential generation of forces that result in the desired athletic function (10). Moreover, adding core training to the training regimen of adolescent handball players may have a positive effect on throwing velocity as being demonstrated by several intervention studies (13, 14). More specifically, Saetterbakken et al. (13) executed a twice a week, sling exercise core stability program consisting of six unstable closed kinetic chain exercises with a group of female high-school handball players (*n* = 24, 16.6 ± 0.3 years) of whom 14 executed the training program as the intervention group and 10 served as a control group. The authors concluded that core stability training can significantly improve maximal throwing velocity. Based on a similar approach, Manchado et al. (14) investigated the effects of a core training intervention program on throwing velocity in 30 handball players (*n* = 16 junior age category, *n* = 14 senior category). For ten weeks, the intervention group participated in a 7 exercises core program performed after the general warm-up in each regular session (four times a week). The authors reported statistically significant differences (*p* ≤ 0.05) in throwing velocity between the experimental group (+4.5%) and the control group (−0.4%).

Based on the literature discussed, it can be hypothesized that core strength is a prerequisite to allow optimal production, transfer, and control of force to the extremities (15). Additionally, it can be hypothesized that an association between core muscle strength endurance and upper-extremity performance, here throwing velocity, can be expected.

To the best of our knowledge, only one study in handball (16) and a relatively small number of studies in general (17–22) assessed the associations between core muscle strength and upper-extremity performance both on a general as well as on a sport-specific level. Precisely, Tomasa et al. (16) assessed the relationship between strength in trunk flexion and throwing velocity in sixteen female elite-level handball players (19.9 ± 3.1 years) from Norway. Contrary to their hypothesis, the authors found no significant association between measures of trunk flexion strength and throwing velocity in either the standing throw with run up nor the jump throw. The authors concluded that trunk flexion strength is not a differentiating factor for throwing velocity in elite women players. However, studies from other sports and populations reported varying results ranging from positive over not significant to negative correlations between core muscle strength and upper-extremity performance. For instance, Westrick et al. (18) found positive relationships between core muscle strength and the Upper Quarter Y Balance (YBT-UQ) test in members from a United States Military Academy. Moreover, Nuhmani et al. (22) in collegiate athletes reported positive associations between core muscle strength and upper-extremity performance (i.e., YBT-UQ test, medicine ball throw test). On the contrary, negative correlations between core muscle strength endurance and upper-extremity performance (i.e., bat swing velocity) were reported by Lin et al. (19) for male high school baseball players. Lastly, no correlations between core muscle strength and upper-extremity performance (i.e., YBT-UQ test) were shown by Bullock et al. (20) in collegiate pitchers and Ahmed et al. (21) in male professional badminton players for core muscle strength and the medicine ball throw test. Additionally, Okada et al. (17) did not find a relationship between core muscle strength and different performance parameters of the Functional Movement Screen in healthy individuals. Based on these findings it is reasonable to assume task-specific associations between core muscle strength endurance and upper-extremity performance in trained individuals. As knowledge regarding possible relationships and interactions may help practitioners to tailor core strength exercises to the needs of a particular sport, sport-specific studies are needed. To the best of our knowledge, no study so far assessed the associations between core muscle strength and shoulder mobility/stability as well as the sport-specific throwing performance in adolescent male sub-elite handball players.

Based on the findings of other studies (18, 22) that reported correlations between core strength and performance parameters we hypothesized that correlations existed between core strength and (a) the performance parameter (YBT-UQ) and (b) the sport-specific parameter (throwing velocity) in handball players.

Therefore, the purpose of the study was to assess whether associations between core muscle strength endurance and (a) shoulder mobility/stability as well as (b) throwing velocity in adolescent male sub-elite handball players exist. We hypothesized that better core muscle strength endurance correlated with (a) better shoulder mobility/stability and (b) higher throwing velocity.

## Methods

### Participants

Using G^*^Power (23), an a priori power analysis (ρ H1 = 0.5, α = 0.05, 1-β = 0.80, ρ H0 = 0) was conducted and revealed that a total sample size of *N* = 29 participants would be sufficient to detect statistically significant Pearson‘s correlations.

Thirty-two male adolescent handball players of two teams which competed in the same regional sub-elite playing class took part in the study (Table 1). All players under 18 years of age handed in an informed consent from their parents or legal guardians whereas the players aged 18 years and above handed in a written consent. Players were excluded when they reported an illness or an actual or recent injury that was judged to potentially have an influence on the results of either test. The study was carried out according to the Declaration of Helsinki and the Human Ethics Committee at the University of Duisburg-Essen approved the study protocol (TM_23.03.2020).

**Table 1:** Characteristics of the study participants (*N* = 32).

**Characteristic**	**Value**
Age [years]	17.1 ± 0.7
Body mass [kg]	81.4 ± 18.0
Body height [cm]	181.8 ± 6.3
BMI [kg/m^2^]	24.6 ± 4.9
Throwing arm length [cm]	93.0 ± 3.8
Non-throwing arm length [cm]	92.6 ± 3.7
Training experience [years]	8.5 ± 3.3

Data are group mean values ± standard deviations. BMI, body mass index.

### Testing procedures

#### Assessment of anthropometric variables

The anthropometric variables body height, body mass, and upper-limb length were assessed prior to the first specific testing. Upper-limb measurement was carried out from the distal tip of the middle finger with the shoulder being in 90-degree abduction (24) to the seventh cervical spinous process (C7). A Seca 217 (Seca, Basel, Switzerland) linear measurement scale was used to measure body height with the subjects standing straight without shoes to the nearest 0.1 cm. For the assessment of body mass, a Seca 803 (Seca, Basel, Switzerland) electronic scale was set up to the nearest 100 g with the participants wearing only light sportswear and no shoes. The body mass index (BMI) was calculated based on the formula: Body mass divided by the measured body height squared (kg/m^2^). Additionally, all subjects were asked for their training experience in years, their dominant arm, the writing arm determined by self-report which writes as well as their throwing arm.

For each of the tests described in the following, all participants had a short familiarization trial before executing the actual tests. In order to avoid fatigue, only one repetition of the final execution was performed during the trials to give the participants the chance to accustom themselves with the tests.

#### Assessment of core muscle strength endurance

**Figure 1:**
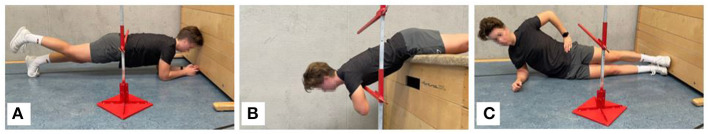
Participant performing the **(A)** ventral, **(B)** dorsal, and **(C)** lateral (right side) chain of the Bourban test.

Core muscle strength endurance was tested based on the Bourban test (Figures 1A–C) which assesses the three motion planes (ventral, dorsal, and lateral). While the ventral and lateral chain movements were performed on a fitness mat, the dorsal chain was tested on a long box.

#### Bourban test: Ventral chain

The ventral chain (Figure 1A) was tested with the subjects being placed in a prone bridge position with straight legs, facing down with parallel under-arms in a vertical upper-arm position and thumbs being upright (25). A straight line from the lateral malleolus to the trochanter major, glenohumeral joint and the greater trochanter was mandatory. At the subject‘s lower back an adjustable alignment device was put into contact at the level of the iliac crest (26). This device consisted of a stable vertical pole and two vertically adjustable horizontal rods (27). Starting from this position, the subjects had to alternately lift their feet about 2–5 cm from the floor to the beat of a metronome (60 bpm – 1-sec lifting, 1-sec lowering). The maximum number of seconds until failure was noted down on the scoring sheet as soon as the subjects a) lost contact to the bar for longer than one second, b) could not keep up with the pace of the metronome or c) could not hold up the requested straight position. The test of the ventral chain can be classified as reliable with a coefficient of variation (CoV) of 14.1% (28) in terms of absolute reliability (29).

#### Bourban test: Dorsal chain

The dorsal chain (Figure 1B) was tested laying prone on a long box with the trunk not being supported (from the upper border of the iliac crest) (26). Arms had to be held across the chest with hands being rested on the shoulders. While the feet had to be firmly fixed within the wood bars behind the box at the wall, the legs had to be fully extended (26). A mechanical goniometer assured the horizontal positioning during the whole execution. A horizontal upper reference rod was fixed at the height of the thoracic spine (26). The subjects had to lower the trunk by 30° again being controlled for by the mechanical goniometer until the lower horizontal reference rod of the alignment device was touched with the sternal angle. A metronome (60 bpm – 1-sec lifting, 1-sec lowering the trunk) was used as an auditive reference for the subjects to continuously lift and lower the trunk. As soon as the subjects failed to touch the upper or lower horizontal rod (1) for two consecutive times or (2) a total number of three times, the test was stopped. The best trial (maximum number of seconds) was noted down and used for further analysis. Based on the recommendations regarding absolute reliability (29), a CoV of 11.7% (28) was reported for the dorsal chain assessment.

#### Bourban test: Lateral chain

A bridge position with the legs being extended and the upper foot placed on top of the lower foot was the starting point for the test of the lateral chain (Figure 1C). The supporting shoulder had to be held superior to the respective elbow (26), while the supporting forearm had to be placed flat on the floor with the upper arm being held akimbo. Hips had to be raised until a straight line was reached from the ankles up to the shoulders. The lower horizontal reference rod of the alignment device was fixed at the height of the superior iliac crest (26). The subjects had to continuously raise and lower their hips which was controlled for by a metronome (60 bpm – 1-sec lifting, 1-sec lowering). It was prohibited to touch the floor while the body was in the lowering phase. The test was stopped as soon as the subjects did not fulfill their task for two consecutive times as a warning was always given when an execution was faulty or a number of three times of faulty executions in total. The maximum number of seconds (i.e., the best trial) was used for further analysis. Both sides were tested with a short break in-between. The lateral chain test can be classified as reliable with a CoV of 14.6% (28) based on the recommendations regarding absolute reliability of Stokes et al. (29).

#### Assessment of shoulder mobility/stability

**Figure 2:**
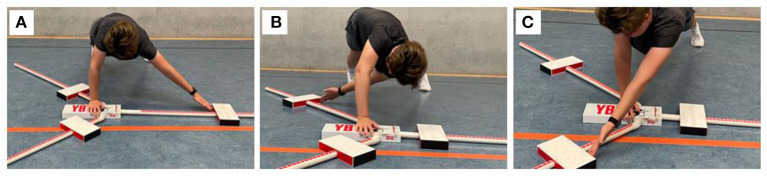
Participant performing the **(A)** medial, **(B)** inferolateral, and **(C)** superolateral reach direction of the Upper Quarter Y-Balance test.

The YBT-UQ (Figures 2A–C) was assessed with a Y Balance Test Kit (Move2Perform, Evansville, IN). An adapted YBT-UQ testing protocol was used during the trials. Before starting, one of the examiners practically demonstrated the correct execution of the YBT-UQ and also delivered a verbal instruction. The feet had to be placed shoulder wide apart while all subjects started in the push-up position (24). With the right arm always being the first stance arm, the indicator had to be consecutively moved to the medial (MD), inferolateral (IL), and superolateral (SL) reach direction without a break. After the three trials were executed with the right arm as the stance arm and the left arm as the mobile arm, the subjects had to change the stance arm to the left arm with the right arm being the mobile arm. Trials were invalid when the subjects did not maintain the push-up position with the contralateral arm as the stance arm on the test device or if the subjects actively pushed the indicator (i.e., lost contact before the final position). Additionally, a three-point contact on the floor had to be maintained at any time during the trials (left and right foot on the floor, stance arm on the device). A rest period of 30-sec was granted between the trials of the right-hand side and of the left side being tested. The values for every trial and reach direction was noted down but only the best score of each reach direction was taken for further analyses (24). The composite score (CS) was also calculated as the mean of the averaged maximal distances of the best trials for all three reach directions normalized for upper-limb length which was measured during the anthropometric assessment.

#### Assessment of throwing velocity

**Figure 3:**
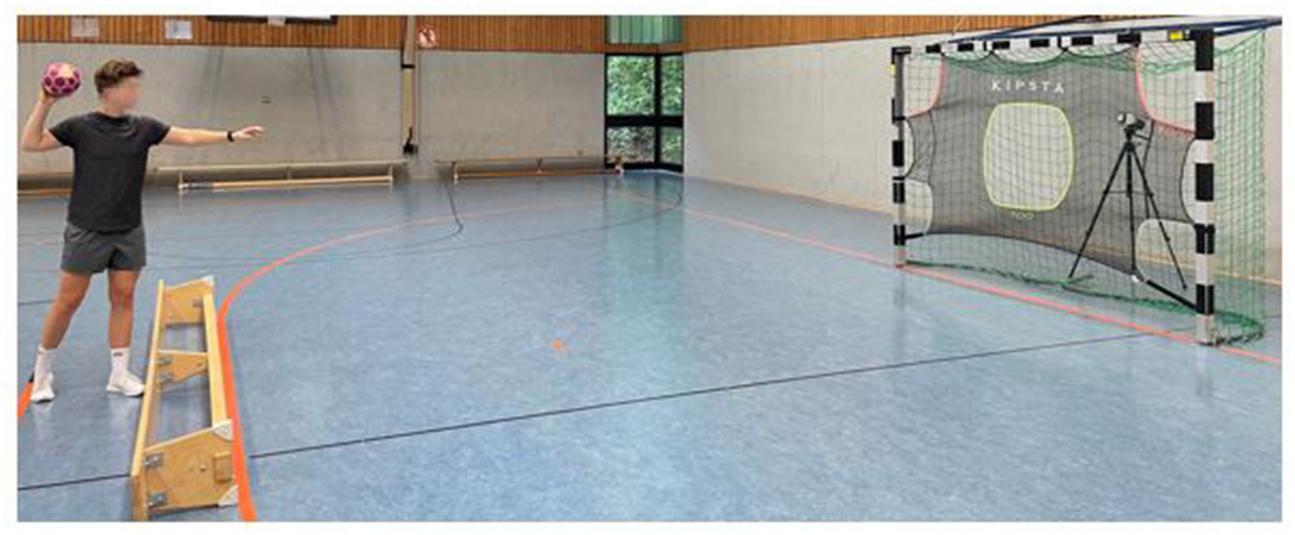
Participant performing the 7-m standing throw test.

A SG 500L target net (3 × 2 m) was attached to a regular handball goal in a training hall. Behind a 1m x 1m hole in the middle of the target net, the “Stalker Pro” radar gun (Applied Concepts Inc., Richardson, TX, USA) was positioned to secure the Doppler effect while the participants were throwing (30). The stalker was fixed at a height of 1.20 m measuring velocities of 0 to 480 km/h with an accuracy of 0.16 km/h in a 0.01 s time interval with a working frequency of 35.1 GHz and a low disturbance threshold (31). Two testers executed the assessment with one tester behind the stalker reporting the values to the second tester who noted them down into the scoring sheet. A standard ball size 3 was used for all attempts. The players had the requirement to position the contralateral leg behind the 7-m line on which a bench was put alongside to assure that the players could not move forward during their throwing attempts. The throws were executed as 7-m standing throws only with the throwing arm with no initial step following the guidelines of the German Handball Association (30) with the instruction to throw as hard as possible into the target net (Figure 3). A short rest was granted between each throwing attempt and the highest throwing velocity out of the three throws was used for further analyses. Chirosa-Ríos et al. (32) reported this setting as highly reliable (ICC = 0.89) to assess throwing velocity.

### Statistical analyses

Descriptive statistics were calculated for group mean values and standard deviations using Statistical Package for Social Sciences version 28.0 (IBM Corp., Armonk, NY, USA) which was used for all analyses. Pearson‘s correlations with 95% confidence interval were calculated for the associations between the four conditions of core strength (Bourban test: ventral chain, dorsal chain, lateral chain - left side, lateral chain - right side) and (a) shoulder mobility/stability (YBT-UQ throwing and non-throwing arm reach) and (b) throwing velocity. The alpha value was *a priori* set at *p* < 0.05 for all tests.

## Results

Table 2 shows the descriptive statistics for measures of core muscle strength endurance and upper-extremity performance.

**Table 2:** Descriptive statistics for measures of core muscle strength endurance and upper-extremity performance.

**Test**	**Value**
**Core muscle strength endurance**	
Bourban test: ventral chain [s]	81.8 ± 32.7
Bourban test: dorsal chain [s]	60.3 ± 21.8
Bourban test: lateral chain, right side [s]	48.2 ± 19.3
Bourban test: lateral chain, left side [s]	50.4 ± 20.5
**Upper-extremity performance**	
**Throwing arm reach**	
YBT-UQ test: MD [% AL]	111.2 ± 9.6
YBT-UQ test: IL [% AL]	103.1 ± 14.9
YBT-UQ test: SL [% AL]	82.4 ± 14.2
YBT-UQ test: CS [% AL]	98.9 ± 10.9
**Non-throwing arm reach**	
YBT-UQ test: MD [% AL]	110.2 ± 9.3
YBT-UQ test: IL [% AL]	104.3 ± 13.7
YBT-UQ test: SL [% AL]	81.2 ± 13.0
YBT-UQ test: CS [% AL]	98.6 ± 10.1
Throwing velocity [km/h]	77.1 ± 7.2

Data are group mean values ± standard deviations. AL, arm length; CS, composite score; IL, inferolateral reach direction; MD, medial reach direction; SL, superolateral reach direction; YBT-UQ, Upper Quarter Y Balance test.

The Bourban test is a well-established test which compromises a standardized set of three dynamic tests of the ventral, lateral, and dorsal trunk muscle chain (33). Likewise, the YBT-UQ places demands on core muscle endurance when maintaining the one arm push-up position while assessing upper extremity mobility/stability (34). Throwing velocity was chosen as the parameter that reflects sport-specific performance as higher throwing velocity increases the likelihood to score (12).

### Correlations between core muscle strength endurance and shoulder mobility/stability

**Figure 4:**
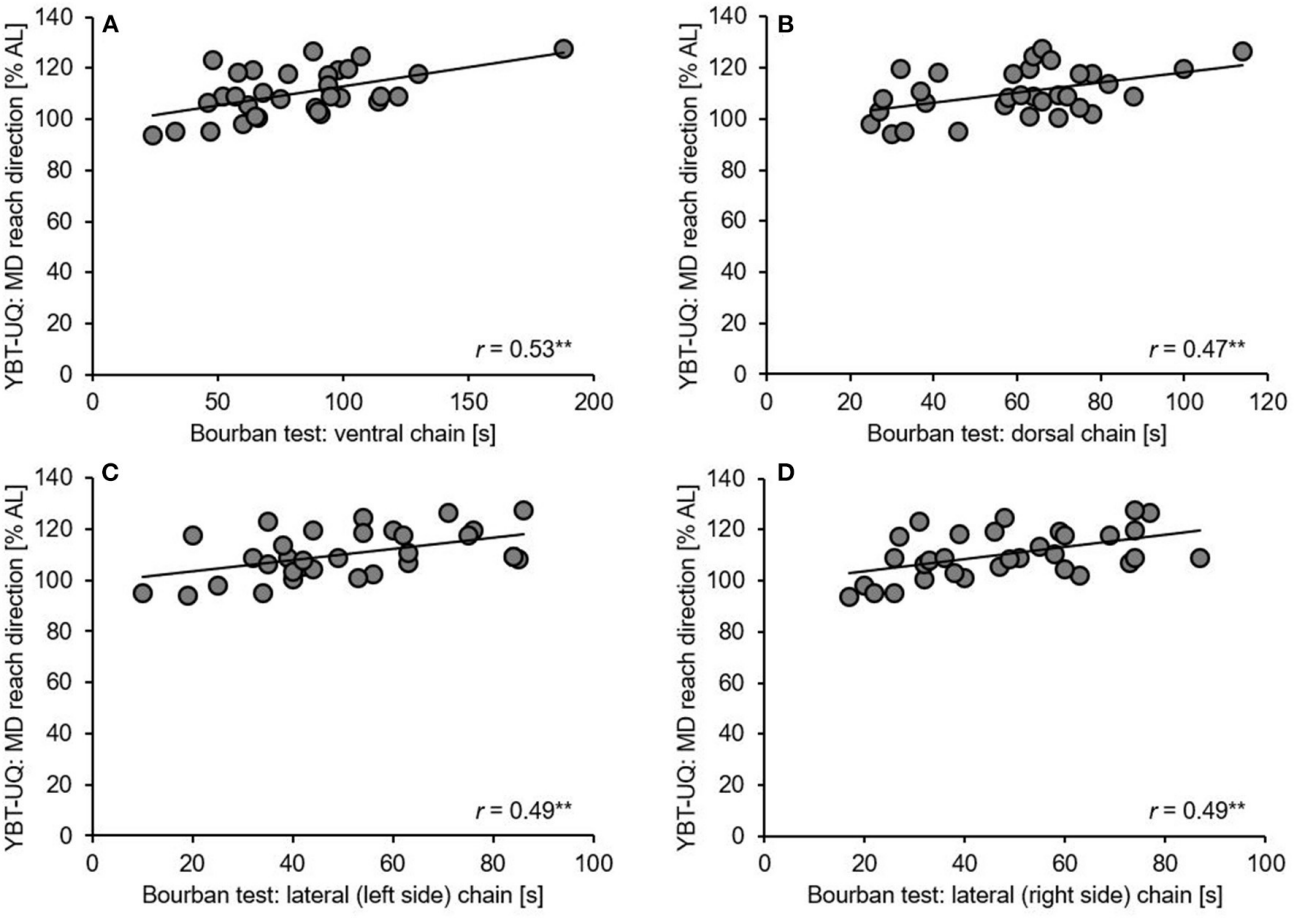
Pearson‘s correlations between the medial (MD) reach direction of the Upper Quarter Y-Balance (YBT-UQ) test and the **(A)** ventral, **(B)** dorsal, **(C)** lateral (left side), and **(D)** lateral (right side) chain of the Bourban test. **correlation is significant at the 0.01 level (1-tailed).

Correlations between core muscle strength endurance (i.e., Bourban test) and shoulder mobility/stability (i.e., YBT-UQ test) are displayed in Table 3 and Figures 4A–D.

**Table 3:** Pearson correlations with 95% confidence interval between core muscle strength endurance and upper-extremity performance (i.e., shoulder mobility/stability and throwing velocity).

	**Bourban test: ventral chain [s]**	**Bourban test: dorsal chain [s]**	**Bourban test: lateral chain, left side [s]**	**Bourban test: lateral chain, right side [s]**
**Shoulder mobility/stability**				
**Throwing arm reach**				
YBT-UQ test: MD [% AL]	0.24 (−0.11–0.55)	0.42** (0.09–0.67)	0.23 (−0.13–0.54)	0.24 (−0.12–0.55)
YBT-UQ test: IL [% AL]	0.41* (0.07–0.66)	0.61** (0.33–0.79)	0.23 (−0.13–0.54)	0.40* (0.06–0.66)
YBT-UQ test: SL [% AL]	0.20 (−0.16–0.51)	0.26 (-0.10–0.56)	0.25 (−0.11–0.55)	0.21 (−0.15–0.52)
YBT-UQ test: CS [% AL]	0.34* (−0.1–0.62)	0.51** (0.20–0.73)	0.28 (−0.08–0.57)	0.35* (−0.01–0.62)
**Non-throwing arm reach**				
YBT-UQ test: MD [% AL]	0.53** (0.22–0.74)	0.47** (0.14–0.70)	0.49** (0.17–0.72)	0.49** (0.17–0.72)
YBT-UQ test: IL [% AL]	0.28 (−0.08–0.57)	0.44** (0.10–0.68)	0.35* (−0.01–0.62)	0.35* (−0.01–0.62)
YBT-UQ test: SL [% AL]	0.05 (−0.31–0.39)	0.17 (−0.19–0.49)	0.14 (−0.22–0.57)	0.14 (−0.22–0.47)
YBT-UQ test: CS [% AL]	0.31* (−0.5–0.59)	0.41** (0.08–0.67)	0.37* (0.02–0.64)	0.37* (0.02–0.64)
**Sport-specific technique**				
Throwing velocity [km/h]	0.18 (−0.19–0.50)	0.33* (−0.03–0.61)	0.20 (−0.17–0.52)	0.29 (−0.07–0.59)

^*^correlation is significant at the 0.05 level (1-tailed); ^**^correlation is significant at the 0.01 level (1-tailed). AL, arm length; CI, confidence interval; CS, composite score; IL, inferolateral reach direction; MD, medial reach direction; SL, superolateral reach direction; YBT-UQ, Upper Quarter Y Balance test.

For the throwing arm reach, we detected significant positive correlations (all *p* < 0.05) between the ventral chain and the IL reach direction (*r* = 0.41) as well as the CS (*r* = 0.34). Further, significant positive correlations (all *p* < 0.01) were found for the dorsal chain with the MD (*r* = 0.42) and the IL (*r* = 0.61) reach direction as well as the CS (*r* = 0.51). Lastly, significant positive correlations (both *p* < 0.05) were obtained for the IL reach direction (*r* = 0.40) and the CS (*r* = 0.35) with the right but not the left side of the lateral chain.

For the non-throwing arm reach, we observed significant positive correlations between the ventral chain and the MD reach direction (*r* = 0.53, *p* < 0.01) as well as the CS (*r* = 0.31, *p* < 0.05). For the dorsal chain, significant positive correlations (all *p* < 0.01) were found for the MD (*r* = 0.47) and the IL (*r* = 0.44) reach direction as well as the CS (*r* = 0.41). Regarding the lateral chain, significant positive correlations were detected for the MD (left and right side: *r* = 0.49, *p* < 0.01) and the IL (left and right side: *r* = 0.35, *p* < 0.05) reach direction as well as the CS (left and right side: *r* = 0.37, *p* < 0.05).

### Correlations between core muscle strength endurance and throwing velocity

**Figure 5:**
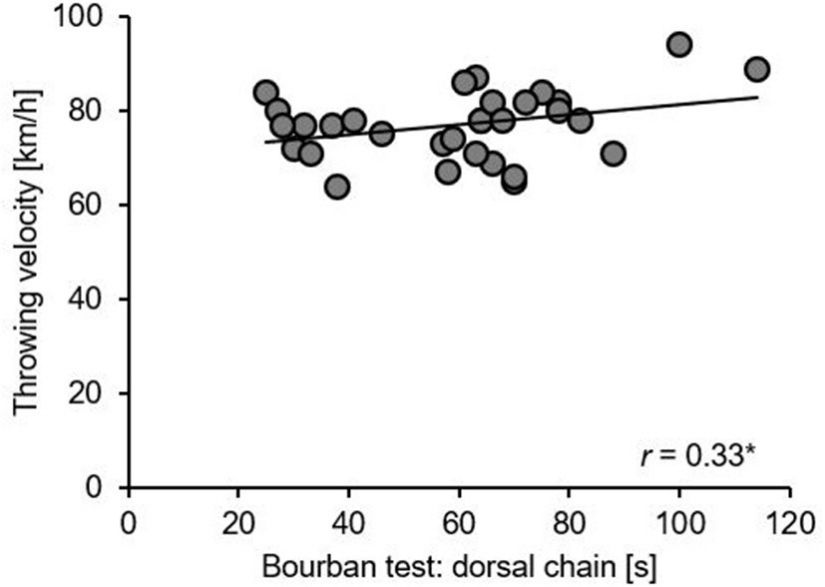
Pearson‘s correlations between throwing velocity of the 7-m standing throw test and the dorsal chain of the Bourban test. *correlation is significant at the 0.05 level (1-tailed).

Correlations between core muscle strength endurance (i.e., Bourban test) and throwing velocity are illustrated in Table 3 and Figure 5. Only, the dorsal chain (*r* = 0.33, *p* < 0.05) but none of the other chains showed a significant positive correlation with throwing velocity.

## Discussion

The aim of the study was to assess correlations between core muscle strength endurance and (a) shoulder mobility/stability as well as (b) throwing velocity in adolescent male sub-elite handball players. The main results can be summarized as follows: Firstly, higher core muscle strength endurance is associated with higher shoulder mobility/stability. Secondly, higher core muscle strength endurance is partially associated with higher throwing velocity.

### Association between core muscle strength endurance and shoulder mobility/stability

The finding that higher core muscle strength endurance is associated with higher shoulder mobility/stability goes in line with Westrick et al. (18) who reported significant positive relationships of YBT-UQ performance with dominant (*r* = 0.38, *p* = 0.04) and non-dominant (*r* = 0.45, *p* = 0.01) side lateral trunk endurance. In accordance, Nuhmani et al. (22) found significant positive correlations between the McGill Score and the YBT-UQ (*r* = 0.46, *p* = 0.01) as well as the MBTT (*r* = 0.67, *p* = 0.02). The present associations may be explained by the fact that the Bourban test is an all-out test that is performed until continuation is no longer possible therefore requiring good level of strength endurance which is also needed during the YBT-UQ. To develop high values of shoulder mobility/stability during the YBT-UQ, the stance arm needs to hold the weight of the person executing the test in a one-arm push-up position as long as the mobile arm is active (35) therefore also emphasizing partly the same endurance-oriented requirements as the Bourban test. On a neuromuscular level, the core muscles are suggested to be innervated in a feed-forward manner during upper-extremity movements (36, 37) enabling sequential generation of power starting from the core and enabling dynamic movements of the extremities as being required during the YBT-UQ. Additional support for this explanation comes from Vasseljen et al. (38) who also suggested the trunk stabilizers to be a feed-forward system which contract prior to the movement of the extremities therefore enhancing stability of the movement. High levels of shoulder mobility/stability may only be possible following an activation of the core muscles which might therefore be interconnected with the upper-extremity performance during the YBT-UQ and the Bourban test. In this line of thought, Hodges (39) suggested that the same synergistic muscle activation patterns especially of large cross-sectional area muscles which recruit the transverse abdominus, abdominals, multifidi, and pelvic floor muscles also provide a base of support for all the trunk and spinal muscles.

During the Bourban test and the YBT-UQ (except for the dorsal chain), the feet provide a stable base of support on the floor enabling this transfer in a similar closed kinetic chain. Consequently, the improved transfer of force may not only be helpful for developing movements of the extremities but also in developing these movements at the end range of mobility and stability as being required during the YBT-UQ. This may be especially true as the final movements of the YBT-UQ are performed in an increasingly fatigued state which is also the case in the strength endurance-oriented Bourban test.

### Association between core muscle strength endurance and throwing velocity

Out of the four core muscle chains (i.e., ventral, dorsal and lateral - left and right side) only the dorsal chain showed a significant positive correlation with throwing velocity. As a possible explanation, it is the dorsal chain which is responsible for trunk extension. On a kinematic level, extension of the trunk in the sagittal plane is important in the initial phase of the throwing movement with the core being the power generator, whereas the arms, acting as the extension of the trunk, are directing the force output to the ball (10). As core muscle strength may be a prerequisite to allow for higher shoulder mobility/stability enabling larger arm cocking is reported to have a positive effect on ball velocity (12, 40, 41). In this context, it was shown that the internal rotation movement starting from a maximal external rotation angle is one of the main contributors for developing force in overarm throwing in team handball (11, 42). Additionally, the same muscles which are recruited during the dorsal chain of the Bourban test, i.e., the quadratus lumborum and the erector spinae as well as the antagonists, rectus abdominis and transversus abdominis at the front of the core may also contribute to the development of power during throwing (43). However, the remaining chains of the Bourban test do not seem to be specific enough to have a significant positive association with throwing velocity which goes in line with Van Den Tillaar and Ettema (12) who also did not find any correlation between throwing velocity and trunk strength.

### Limitations

There are some limitations that need to be addressed. As the present study is a correlational study, potential effects of improving core strength on throwing velocity were not examined. Moreover, the present results can only be transferred to adolescent male sub-elite players. Additionally, the results are only based on the findings of two specific tests. The present results can first and foremost be transferred to core muscle strength endurance as this is the main aspect being tested during the Bourban test. Thus, no comments can be made on the association between maximal strength (i.e., peak force and/or a high rate of force development) and sport-specific or general performance parameters. Throwing actions in handball are a high velocity/fast recruitment rather than a strength endurance task. Therefore, maximum strength-oriented tests may bring added value to test for the associations between core muscle strength endurance and throwing velocity. Additionally, as throwing is a multifactorial task affected by strength, power, mobility, and kinetic aspects among other factors, it may be the case that different players emphasize these factors differently. Moreover, based on the study design only correlations can be reported. However, it remains unclear if these correlations are causal in nature or if for example athletes with a higher core strength, in general do possess higher physical fitness of which shoulder mobility/stability or throwing velocity are only examples of. For the assessment of sport-specific abilities, the standing throw was chosen with the advantage of good standardization and validity. However, the jump throw is much more frequently displayed during games and is known to require different neuromuscular patterns (44) than a 7-m throw or any throwing technique with ground contact. Therefore, the results of the present study do not necessarily reflect associations between core muscle strength endurance and throwing velocity during jump throws. Additionally, only the associations between core muscle strength endurance and upper-extremity performance were assessed. Therefore, it would also be interesting to assess correlations between core muscle strength endurance and lower-extremity performance. In terms of the relationship with throwing velocity this may be especially useful as throwing velocity may be more related to lower-extremity strength and force production (45).

## Conclusion

We investigated the associations between core muscle strength endurance and shoulder mobility/stability as well as throwing velocity in adolescent male sub-elite handball players. Our results indicate that higher core muscle strength endurance is associated with higher shoulder mobility/stability and that especially a higher strength endurance of the dorsal trunk muscle chain is correlated with a higher throwing velocity. As handball players frequently perform motions in shoulder abduction which corresponds with the MD reach direction and external rotation which corresponds with the IL reach direction of the YBT-UQ during throwing, passing and specific defense techniques, these movements may lead to a reduced upper extremity mobility/stability in the throwing shoulder (46). Therefore, practitioners should integrate core strength exercises and/or shoulder mobility/stability exercises into the training routines of handball players to counteract this undesired adaptation as the interrelation of both aspects may have beneficial effects for the other. Additionally, the integration of strength exercises, especially for the trunk muscles of the dorsal chain, may additionally have an improving effect on the important handball-specific throwing velocity. Future studies should investigate the correlations of maximal strength and sport-specific performance parameters as most sport-specific tasks rather rely on maximum strength development than on strength endurance during the specific motor action. In the case of handball, it could alternatively be the jump throw as a sport-specific performance parameter to be tested as this is the much more frequently displayed throw during training and games. Moreover, future studies should investigate if the correlations in the present study are also present in other age groups or female handball players.

## Data availability statement

The raw data supporting the conclusions of this article will be made available by the authors, without undue reservation.

## Ethics statement

The studies involving human participants were reviewed and approved by Human Ethics Committee at the University of Duisburg-Essen. Written informed consent to participate in this study was provided by the participants' legal guardian/next of kin.

## Author contributions

JB and TM designed the research question and analyzed the data. JB planned and supervised the testing, conducted the testing and data collection, and wrote the main part of the manuscript. TM and MG reviewed the manuscript. All authors approved the final manuscript.

## Funding

We acknowledge support by the Open Access Publication Fund of the University of Duisburg-Essen. The funding body is independent of the design of the study and collection, analysis, and interpretation of data and in writing the manuscript.

## Conflict of interest

The authors declare that the research was conducted in the absence of any commercial or financial relationships that could be construed as a potential conflict of interest.

## Publisher's note

All claims expressed in this article are solely those of the authors and do not necessarily represent those of their affiliated organizations, or those of the publisher, the editors and the reviewers. Any product that may be evaluated in this article, or claim that may be made by its manufacturer, is not guaranteed or endorsed by the publisher.

## Abbreviations

AL: Arm length
BMI: Body mass index
CS: composite score
IL: Inferolateral
MD: medial
SL: Superolateral
YBT-UQ: Upper Quarter Y Balance test.
